# Green light modulates disease resistance in *Arabidopsis thaliana* against *Pseudomonas syringae* pv. *tomato*

**DOI:** 10.1080/15592324.2025.2546465

**Published:** 2025-08-20

**Authors:** Swarnalee Dutta, Shamjetsabam Gangarani Devi, Yong Hoon Lee

**Affiliations:** a Division of Biotechnology, Jeonbuk National University, Jeollabuk-do, Republic of Korea; b Advanced Institute of Environment and Bioscience, Plant Medical Research Center, and Institute of Bio-industry, Jeonbuk National University, Jeollabuk-do, Republic of Korea

**Keywords:** Green light, jasmonic acid, light, photoreceptor, resistance

## Abstract

Light plays a pivotal role in regulating plant physiological processes. However, the influence of specific light wavelengths on plant defense to pathogen infection remains insufficiently explored. We investigated the role of different light wavelengths, with a particular focus on green light (GL), in modulating disease responses and signaling in Arabidopsis. We pretreated Arabidopsis Col-0 plants with various light wavelengths before challenging them with *Pseudomonas syringae* pv. *tomato* DC3000 (PstDC3000). GL at an intensity of 100 µmol m^–2^ s^–1^ significantly suppressed disease incidence compared to white light (WL). GL upregulated the expression of key defense-related genes, including *COI1, JAR1, PDF1.2,* and *PAD4, *compared to WL. Furthermore, GL promoted callose deposition and reduced H_2_O_2_ production relative to the WL treatment. Jasmonic acid (JA)-deficient mutants (*∆Jar1* and *∆Coi1*) exhibited heightened disease severity under GL treatment compared with that of Col-0, underscoring the central role of the JA pathway in GL-mediated defense responses. These results indicate that GL functions as a crucial modulator of the defense response in Arabidopsis, offering new insights into the relationship between light quality and plant immunity. However, further research is required to elucidate the precise perception mechanisms and signaling networks involved in GL-mediated defense responses.

## Introduction

Light plays a pivotal role in the regulation of numerous physiological and developmental processes in plants, including defense mechanisms against pathogens.^1,2^ Plants utilize photoreceptors to perceive different light spectra, enabling them to modulate their defense strategies.^3^ Arabidopsis perceives light signals through different families of photoreceptors, namely red and far-red light-sensing phytochromes (PHYA–PHYE), blue light/UV-A-sensing cryptochromes (CRY1–CRY3), phototropins (PHOT1 and PHOT2), and zeitlupes (ZTL, FKF1, and LKP2).^1,4-6^

Light quality, intensity, duration, and timing critically influence plant defense signaling against pathogens.^7-10^ Specific wavelengths can trigger distinct defense responses in Arabidopsis. For instance, light perception via phytochromes induces PR-1 accumulation and a hypersensitive response (HR) in Arabidopsis during incompatible interactions with the turnip crinkle virus.^11^ Red light (RL) induces systemic disease resistance in Arabidopsis against PstDC3000 and the root-knot nematode *Meloidogyne javanica.*^12^

Salicylic acid (SA) and jasmonic acid (JA) are important signaling molecules involved in plant defense responses to various types of biotic stress. JA predominantly mediates responses to necrotrophic pathogens and herbivorous insects by triggering transcription factors regulating JA-responsive genes.^13-15^ SA leads to the systemic expression of defense-related genes, such as *PR-1a*, and protects plants from secondary invasion by pathogens.^15^ Light modulates the production of both JA and SA, thereby influencing defense responses.^16^^,^^17^ Photoreceptors and transcription factors involved in phototransduction are connected to the SA and JA signaling pathways, affecting the trade-off between growth and defense.^7^^,^^16^

Studies have highlighted GL as a potent inducer of plant immunity, enhancing disease resistance in tomato plants by upregulating key defense-related genes such as phenylalanine ammonia-lyase (PAL) and pathogenesis-related protein 1a (*PR-1a*).^18^ Additionally, GL promotes the production of antibacterial substances and reinforces cell walls through lignin deposition.^19^ Ding et al.^20^ demonstrated the impact of continuous global low-intensity GL on tobacco (*Nicotiana tabacum*) plants, revealing physiological responses, such as necrotic spot formation, altered ion dynamics, and reactive oxygen species (ROS) production. However, the precise molecular mechanisms and signaling pathways governing the influence of monochromatic light on plant defenses remain poorly understood. Here, we investigated the effects of monochromatic RL, GL, and blue light (BL) on disease incidence in Arabidopsis challenged with PstDC3000. We analyzed GL-mediated defense mechanisms by examining gene expression, callose deposition, and hydrogen peroxide (H₂O₂) production, focusing on the key components of the JA and SA signaling pathways. Additionally, we used Arabidopsis mutant lines with disrupted signaling pathways to assess the contribution of specific genes to GL-induced disease resistance against PstDC3000 infection. Our findings revealed that GL significantly suppressed disease incidence in Arabidopsis through the regulation of defense-related genes and metabolites. These results highlight the pivotal role of GL in modulating plant defense responses and provide insights into the interaction between light signaling and plant immunity. Further research is essential to elucidate the precise molecular mechanisms and signaling networks underlying GL-mediated defense responses in plants.

## Materials and methods

### Arabidopsis growth conditions

Arabidopsis seeds were sterilized with 70% ethanol for 1 min, treated with 1% sodium hypochlorite for 15 min, and rinsed five times with sterile distilled water (DW). The surface-disinfected seeds were sown in commercial soil (Cham-grow, Korea) in 150 ml pots and grown in a plant growth room maintained at 22 ± 2°C with a 16-h light/8-h dark photoperiod. The relative humidity was maintained at 50%–60%.

### Light treatment systems

Light-emitting diode (LED) light sources were used to emit specific light wavelengths: RL (typical light emission at 645 nm), GL (524 nm), BL (458 nm), and white light (broad spectrum, WL).^18^^,^^21^ LEDs were installed in the upper ceiling of the chambers and connected to a circuit box, allowing control of the intensity of each wavelength. The chambers were equipped with air channels and enclosed with light-impenetrable black walls to block external light. The photosynthetic photon flux density (PPFD), which is quantified as µmol photons m^–2^ s^–1^ from each light source, was measured using a quantum sensor (LI-190 SB; Li-Cor, Lincoln, USA) as needed.

### Disease incidence and in tissue pathogen growth assay

The effects of light wavelength on disease incidence in Arabidopsis following PstDC3000 infection were investigated following the method of Furci et al.,^22^ with minor modifications. Briefly, rifampicin-resistant PstDC3000 was cultured overnight in Luria broth (LB) (OD 0.8–1.0) at 28°C, centrifuged at low speed to collect the bacterial cells, washed twice with 10 mM MgCl_2_ solution, and resuspended to OD_600_ = 0.2 (1 × 10^8^ CFU/ml) in 10 mM MgCl_2_ solution containing 0.025% Silwet L-77. Four- to five-week-old Arabidopsis Col-0 plants were placed in the LED chamber and illuminated with each wavelength (WL, GL, BL, and RL) at an intensity of 100 µmol m^–2^ s^–1^ for 6 h (Figure S1). This pretreatment step was included to ensure a consistent and uniform light environment prior to pathogen challenge and was not intended to assess light-induced priming effects. The leaves of Arabidopsis plants were evenly sprayed with the prepared PstDC3000 suspension and cultivated in the LED chamber under 16-h light/8-h dark conditions at 20°C ± 2°C. The relative humidity was maintained at 90%–100% for 24 h after pathogen inoculation, and disease development was observed for 7 d.^18^ The influences of light intensity on disease incidence were also assessed under the illumination of GL and WL at 75, 100, and 125 µmol m^–2^ s^–1^. PstDC3000-infected Arabidopsis leaves were collected 7 d after inoculation, weighed, and surface-sterilized with 15% H_2_O_2_ followed by rinsing with sterile distilled water (DW). The collected leaves were placed in DW in Eppendorf tubes and homogenized with a pellet pestle, and the suspensions were serially diluted. A 100-µl aliquot of each diluted sample was spread on LB agar plates containing rifampicin (50 µg ml^−1^), and the number of bacterial colonies in each plate was counted 24 h after incubation at 30°C.

Additionally, Arabidopsis mutants deficient in the JA signaling pathway, including jasmonate resistant 1 (*∆jar1*) and coronatine-insensitive 1 (*∆coi1*), and SA signaling pathways like salicylic acid induction deficient 2 (∆*sid2*), and nonexpresser of PR genes (*∆npr1*) were grown in a pot for 4–5 weeks. These mutants were pretreated with GL or WL at an intensity of 100 µmol m^–2^ s^–1^ for 6 h and inoculated with PstDC3000 as described above. The plants were grown under GL or WL conditions, and disease severity was assessed 7 d after inoculation. Disease severity was rated on a scale from 0 to 4, where 0 = no symptoms (healthy leaves) and 4 = completely diseased leaves. Disease severity was calculated using the formula: [∑(class frequency × score of rating class)]/[(total number of plants) × (maximal disease index)] × 100.

### Gene expression analysis using qPCR

Leaves from five individual Arabidopsis plants treated with GL and WL were collected in liquid nitrogen at 0, 24, and 72 h after inoculation and frozen in liquid nitrogen. The samples were homogenized using a mortar and pestle, and total RNA was isolated using the RNAqueous^TM^ Phenol-Free Total RNA Isolation Kit (Invitrogen, USA). cDNA was synthesized from each RNA sample using the TOPscript^TM^ cDNA Synthesis Kit (Enzynomics^TM^, Korea) following the manufacturer's instructions. Gene expression of pathogenesis-related-1a (*PR-1a*), nonexpresser of pathogenesis-related genes 1 *(NPR1*), plant defensin 1.2 (*PDF1.2*), jasmonate resistant 1 (*JAR1*), phytoalexin deficient 4 *(PAD4),* enhanced disease susceptibility 1 (*EDS1*), salicylic acid induction deficient 2 (*SID2*), and coronatine-insensitive 1 (*COI1*), was assayed using QuantStudio^TM^ 1 Real-Time PCR Instrument (Applied Biosystems, USA) with each primer set (Table S1). The PCR reaction mixture, 10 µl of 2× SYBR Green Supermix, 1 µl of diluted cDNA, and 1 µl of each primer were cycled as follows: preliminary denaturation at 95°C for 15 min and 40 cycles of denaturation at 95°C for 15 s, annealing for 30 s at 52°C, and elongation at 72°C for 30 s. The cycle threshold (Ct) values were recorded after the final melting. The specificity of each primer was confirmed by melting curve analysis, and agarose gel electrophoresis was performed using qPCR products. *Actin2* was used as the reference gene for normalization. Relative gene expression was calculated as described by Livak and Schmittgen.^23^ The experiment was conducted in triplicate with three biological replicates.

### H_2_O_2_ accumulation assay

The H_2_O_2_ accumulation was quantified as described by Jack et al.^24^ Leaf samples illuminated with GL and WL were collected 0, 12, and 24 h after pathogen inoculation and extracted in 0.1% trichloroacetic acid buffer. Sample aliquots were mixed with 10 mM potassium phosphate buffer (pH 6.5) and 1 M potassium iodide (1:1:2). The samples were incubated in the dark at room temperature (~25°C) for 20 min, and the absorbance was measured at 390 nm. The values were compared with those of the standard curve for the quantification of H_2_O_2_ in nanomoles. Additionally, H_2_O_2_ accumulation was analyzed by 3,3′-diaminobenzidine (DAB) staining.^25^ Leaves collected at 0, 12, and 24 h after pathogen inoculation were immersed in DAB solution in 12-well plates, followed by gentle vacuum for 5 min. The plates were covered with aluminum foil and slowly rotated at 80–100 rpm for 4 h. The DAB solution was replaced with bleaching solution (ethanol:acetic acid:glycerol in the ratio of 3:1:1), and the plates were incubated in a water bath at 90–95°C for 15 ± 5 min. After replacing the bleaching solution with fresh bleaching solution, the plates were incubated at room temperature for 30 min. DAB staining was visualized against a bright background (Matin Slimlight Panel 5000-L).

### Callose deposition assay

Four- to five-week-old Arabidopsis Col-0 plants were illuminated with GL and WL at an intensity of 100 µmol m^–2^ s^–1^ for 6 h as described above. Leaves of Arabidopsis plants were infiltrated with the prepared PstDC3000 suspension (5 × 10^7^ CFU/ml) and incubated under the respective light conditions in an LED chamber. Infected leaf samples were collected 12 and 24 h after inoculation, and callose deposition was observed following the methods of Scalschi et al.^26^ with minor modifications. Briefly, the collected leaves were cleared in 96% ethanol in 50 ml Falcon tubes until transparent and then rehydrated in sodium phosphate buffer (0.07 M, pH 9) for 30 min. The buffer was discarded and the leaf samples were stained with 0.5% methyl blue overnight in the dark. The leaves were mounted on glass slides with 0.05% methyl blue solution and observed under a fluorescence microscope (Nikon Eclipse Ti2) at 20× magnification using a 475–525 nm filter. Callose deposition was quantified by measuring the percentage area stained with methyl blue in each image using ImageJ software (version 1.54, NIH, USA).^27^ Three biological replicates per treatment were analyzed.

### Statistical analysis

Statistical analyses were performed using the SAS software (Statistical Analysis System 9.2, NC, USA) and Tukey’s test. Student's *t*-test (*P* < 0.05) was used to assess the differences in the effects between WL- and GL-treated plants.

## Results

### Effect of RL, GL, and BL on disease severity and pathogen growth in Arabidopsis

**Figure 1.**
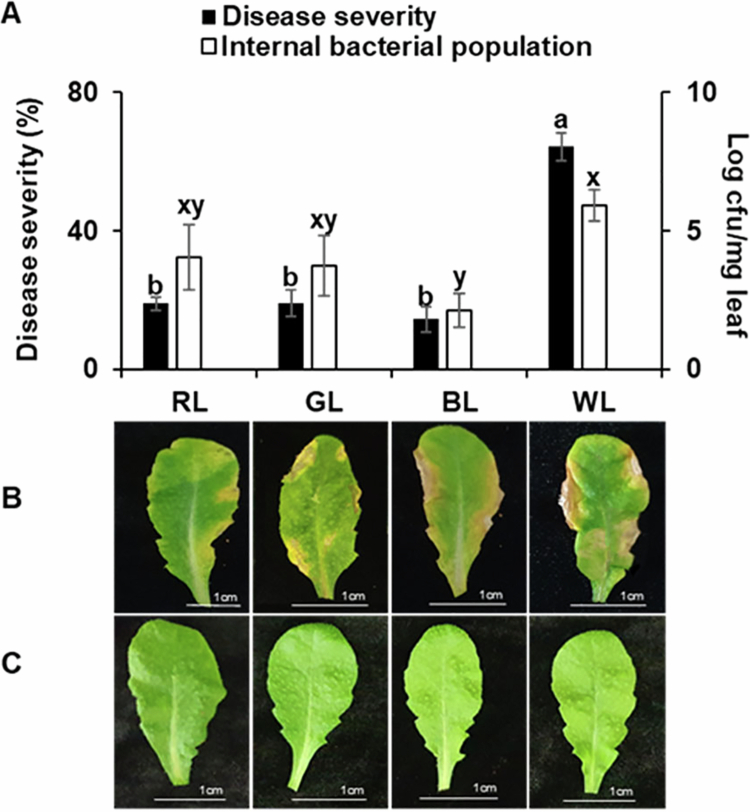
Effect of different light wavelengths on disease severity in Arabidopsis infected by *Pseudomonas syringae* pv. *tomato* DC3000 (PstDC3000).

Our previous study by Nagendran and Lee^18^ demonstrated that light wavelength and intensity significantly influence disease severity in tomato plants infected with *Pseudomonas cichorii* JBC1. Here, we investigated the effects of different light wavelengths on disease severity in Arabidopsis Col-0 following inoculation with PstDC3000. Arabidopsis were exposed to RL, GL, and BL at 100 µmol m^–2^ s^–1^, and disease severity was compared with that of WL. The disease incidence under GL (19.2%), BL (14.5%), and RL (18.9%) was significantly lower than that under WL (64.3%) (Figure 1). Furthermore, bacterial growth in Col-0 tissues was significantly reduced under RL (1.2 × 10^4^ CFU/mg), GL (6.3 × 10^3^ CFU/mg), and BL (1.5 × 10^2^ CFU/mg) compared with those in WL (8.4 × 10^5^ CFU/mg) (Figure 1A). These results revealed the pivotal role of light wavelength in modulating plant defense responses. The observed reductions in disease severity and pathogen proliferation under RL, GL, and BL conditions suggest that manipulating the light environment could be a promising strategy for enhancing plant resistance against pathogens.

### Effects of GL and its intensities on disease severity in Arabidopsis

**Figure 2.**
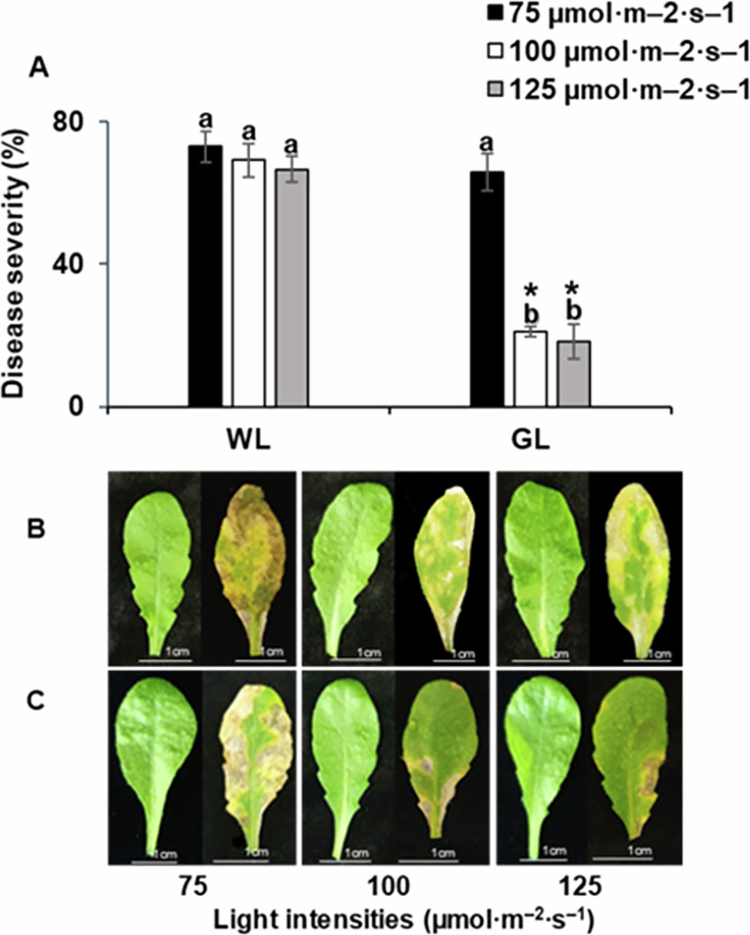
Effect of green and white light intensities on disease severity in Arabidopsis infected by *Pseudomonas syringae* pv. *tomato* DC3000.

To further understand the impact of GL on the interaction between Arabidopsis and PstDC3000, pathogen-inoculated Arabidopsis Col-0 were grown under various GL intensities (75, 100, and 125 µmol m^–2^ s^–1^). Disease severities in Arabidopsis leaves exposed to GL at 75 µmol m^–2^ s^–1^ were comparable to that of the WL control (Figure 2). However, GL at intensities of 100 and 125 µmol m^–2​​​​​^ s^–1^ significantly reduced disease incidence compared with that of WL-treated plants. These results indicate that the intensity of GL plays a critical role in enhancing plant resistance. Specifically, higher GL intensities (100 and 125 µmol m^–2​​​​​^ s^–1^) contribute to reduced disease severity, suggesting a dose-dependent effect of GL on activating defense mechanisms in Arabidopsis.

### Effect of GL on JA-related resistance gene expression

**Figure 3.**
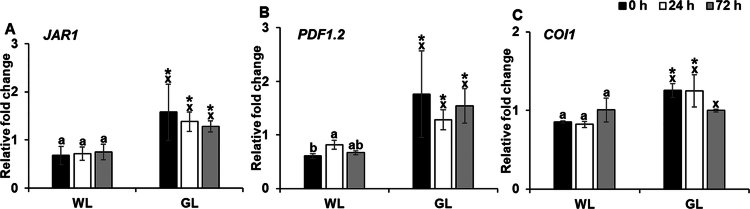
Effect of green light on jasmonic acid signaling pathway gene expression.

JA acts as a key signaling molecule regulating the expression of various genes involved in defense responses to inhibit pathogen growth. To evaluate the influence of GL on JA-mediated defense responses, we analyzed the expression of the JA signaling pathway-related genes *JAR1, PDF1.2*, and *COI1* at 0, 24, and 72 h after challenge with PstDC3000. The expression of both *JAR1* and *PDF1.2* was significantly elevated under GL from the moment of inoculation and was sustained throughout the 72 h period compared with that of WL (Figure 3A,B). *COI1* expression showed a significant increase with GL at 0 and 24 h after inoculation, while at 72h the gene expression was *at par* with WL-treated plants (Figure 3C). While WL treatment resulted in a slight increase in *PDF1.2* expression at 24 h, this was followed by a marked decrease at 72 h (Figure 3B). Overall, the consistent upregulation of *JAR1, PDF1.2,* and *COI1* under GL conditions suggests that GL effectively enhances JA signaling pathways, promoting the sustained activation of JA-dependent defense responses. These results indicate that GL may play a vital role in bolstering JA-mediated resistance against PstDC3000 infection in Arabidopsis.

### Effect of GL on SA-related resistance gene expression

**Figure 4.**
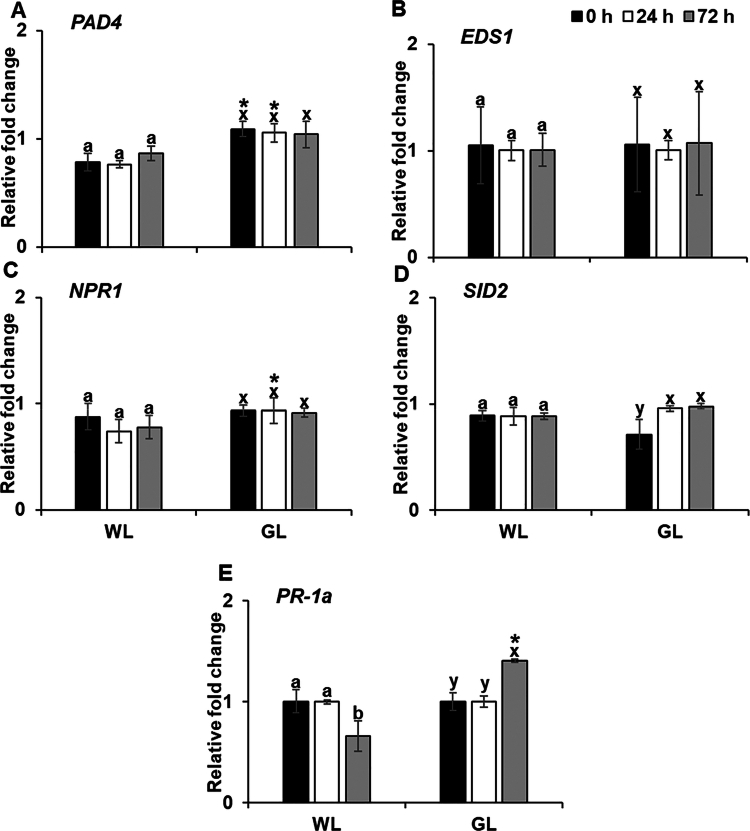
Effect of green light on the expression of salicylic acid signaling pathway genes.

SA induces SAR in plants, including the activation of PR proteins, primarily against biotrophic pathogens. Here, we assessed the expression of key SA-dependent signaling genes, *PAD4*, *EDS1, NPR1, SID2,* and *PR-1a*, which are related to the SA-dependent signaling pathway, at 0 h, 24 h, and 72 h after challenge inoculation. GL treatment initially increased *PAD4* expression compared with that by WL; however, the difference became insignificant at 72 h after treatment (Figure 4A). The expression of *EDS1* remained unaffected by GL at all time points (Figure 4B). Although *NPR1* expression was slightly increased in GL-treated plants at 24 h compared with that in the WL treatment, no significant difference was observed at 72 h (Figure 4C). *SID2* expression showed no substantial variation between the GL and WL treatments at any time point (Figure 4D). Contrastingly, *PR-1a* expression showed a marked divergence. Under GL conditions, *PR-1a* expression significantly increased at 72 h after inoculation, whereas it was suppressed under WL conditions at a later time point. (Figure 4E). This suggests delayed but pronounced activation of *PR-1a* under GL treatment. Overall, while the expression of *PAD4*, *EDS1*, and *NPR1* showed minimal changes under GL compared with those in WL, the late-stage induction of *PR-1a* under GL highlights its potential role in enhancing the SA-mediated defense response in Arabidopsis. The results indicated that GL, apart from a transient increase in *PAD4*, does not broadly impact SA-mediated pathways, although it may specifically enhance *PR-1a*-driven resistance over time in Arabidopsis against PstDC3000 infection.

### Effect of GL on H_2_O_2_ accumulation

**Figure 5.**
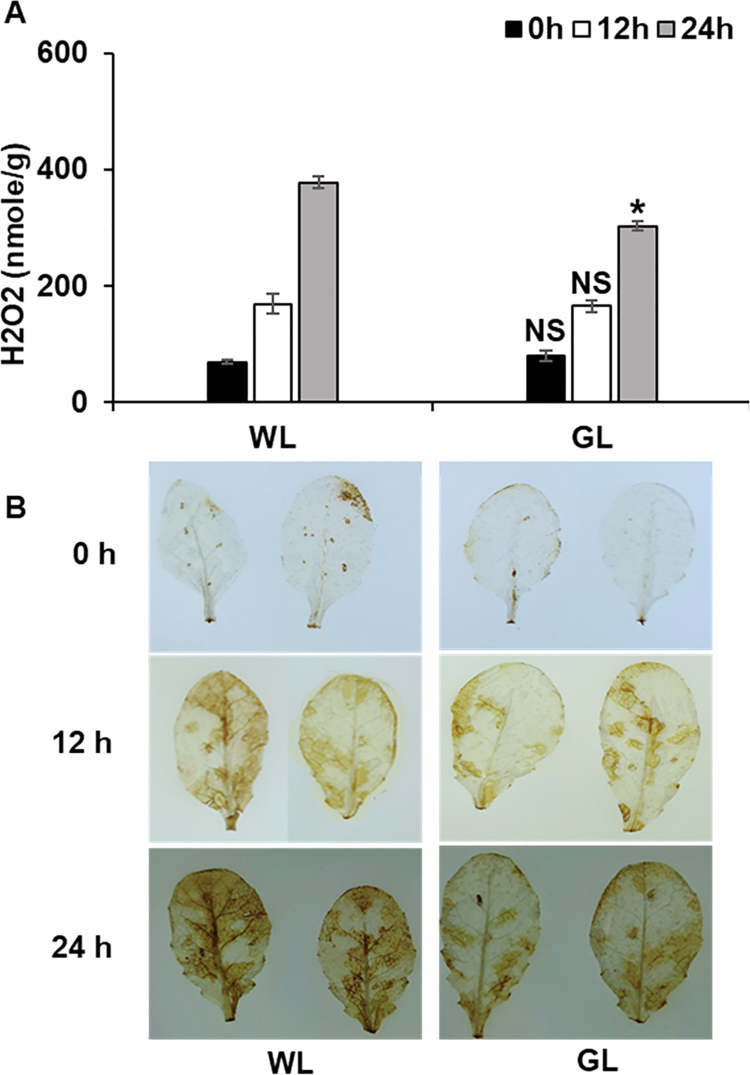
Effect of green light on H_2_O_2_ production and localization.

H_2_O_2_, a critical ROS, plays an essential role in plant responses to abiotic and biotic stresses. To investigate the effects of GL on ROS-mediated defense mechanisms, we quantified H_2_O_2_ production and examined its localization in Arabidopsis leaves treated with GL or WL following challenge inoculation. Quantitative analysis revealed no significant differences in H_2_O_2_ accumulation between the two treatments at 0 and 12 h after inoculation (Figure 5A). However, 24 h after challenge inoculation, H_2_O_2_ levels were significantly lower in GL-treated leaves than in those exposed to WL (Figure 5A).

Additionally, we qualitatively assessed H_2_O_2_ localization by DAB staining. At 0 h, minimal H₂O₂ accumulation was detected (Figure 5B). By 12 and 24 h, WL-treated leaves exhibited more intense DAB staining, indicative of higher H₂O₂ accumulation, whereas GL-treated leaves showed relatively lighter staining, suggesting reduced ROS levels (Figure 5B). These results demonstrate that GL moderates H₂O₂ accumulation in response to pathogen inoculation. The reduction in ROS levels under GL conditions may reflect a fine-tuned balance in oxidative signaling, potentially mitigating ROS-associated damage while maintaining adequate defense responses.

### Influence of GL on callose deposition

**Figure 6.**
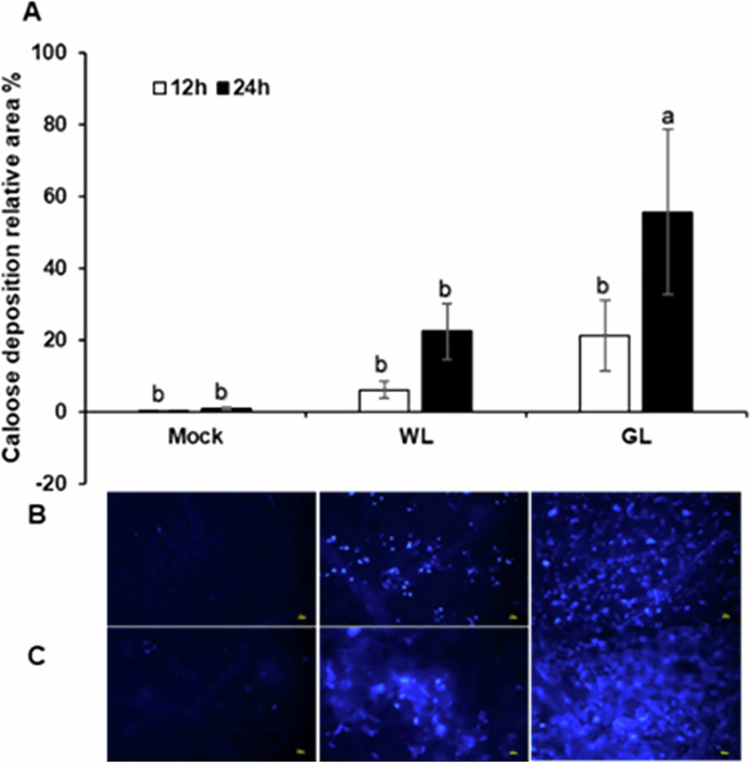
Effect of white and green light on callose deposition.

Callose, a high-molecular-weight β-(1,3)-glucan polymer, reinforces the cell wall and acts as a physical barrier impeding the invasion and spread of pathogens. Here, we investigated the effect of GL on callose deposition in Arabidopsis Col-0 leaves after infiltration with PstDC3000. No significant callose deposition was observed after pretreatment with either GL or WL from 0 to 6 h after inoculation. After 12 h, callose deposition was detectable in both treatments (Figure 6A). At 24 h, the GL-treated leaves exhibited widespread and uniform callose deposition across larger areas of the inoculated leaves (Figure 6B). Although WL-treated leaves also showed increased callose accumulation at 24 h compared with that at 12 h, callose deposition was more localized and primarily concentrated around the infection sites (Figure 6). These results indicate that GL promotes faster, denser, and more extensive callose deposition than those by WL. The enhanced spatial distribution of callose under GL conditions suggests a robust and proactive defense response.

### Effect of GL on disease severities in Arabidopsis mutant lines at JA- and SA-dependent pathways

**Figure 7.**
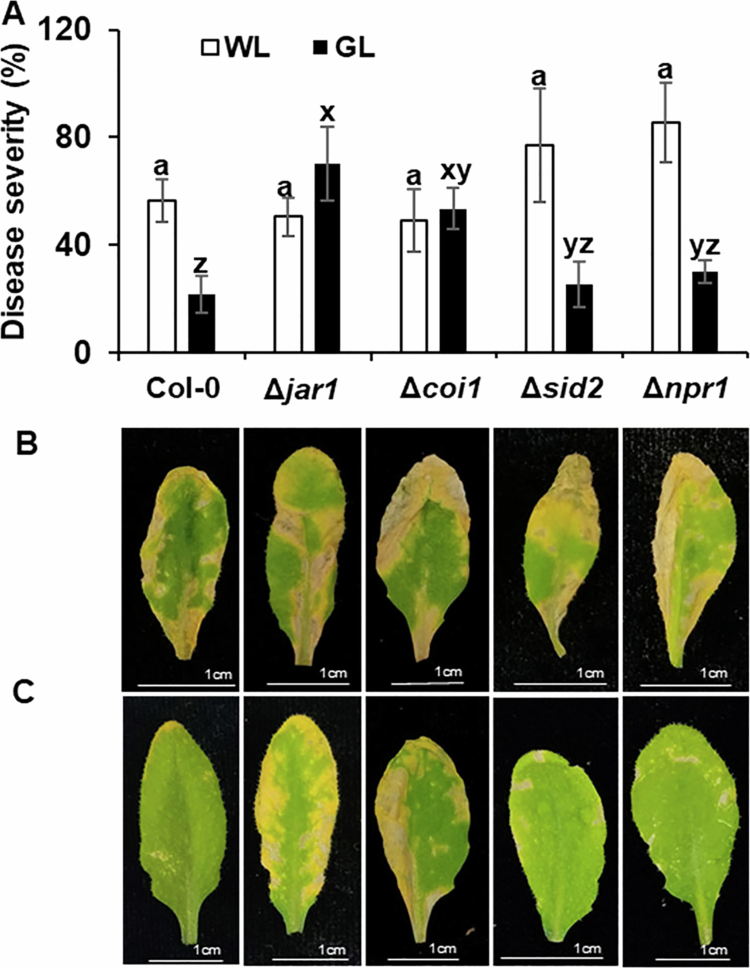
Effect of green light on disease severity in Arabidopsis Col-0 and mutants.

To elucidate the signaling networks influenced by GL and their role in modulating the immune system in Arabidopsis, we examined disease severity in Arabidopsis mutants with impaired SA- and JA-dependent pathway genes following inoculation with PstDC3000. Arabidopsis mutants with impaired JA-related genes (*JAR1* and *COI1*) exhibited significantly higher disease severity under GL conditions than that by Col-0 plants (Figure 7A,C). This aligns with the gene expression data, indicating the upregulation of *JAR1* and *COI1* in response to GL, highlighting the importance of these genes in mediating GL-induced resistance (Figure 3A,C). Contrastingly, mutants impaired in SA-related genes (*NPR1* and *SID2*) showed no significant difference in disease severity under GL conditions compared with that of Col-0 (Figure 7A,C). All the mutants exhibited similar disease severities under WL conditions (Figure 7A,B), confirming that GL uniquely modulates JA-related pathways to influence immune responses. These results suggest that GL enhances resistance primarily through the JA-dependent signaling pathway, as evidenced by the increased susceptibility in JAR1 and COI1 mutants. The lack of significant differences in the *NPR1* and *SID2* mutants under GL conditions further supports the notion that the influence of GL on plant immunity is predominantly JA-mediated. This highlights the potential of GL as a targeted approach for enhancing JA-driven defense mechanisms in plants.

### Networks of defense signaling mediated by GL illumination

Light is a critical regulator of plant immune responses and intricately influences defense mechanisms against pathogenic infections. This study demonstrates the critical role of GL in enhancing the resistance of Arabidopsis to PstDC3000 infection. Using disease severity assays and gene expression analyses, we found that GL preferentially activated the JA-mediated defense pathway (Figure S2). The early and sustained activation of *JAR1* and *PDF1.2* from 0 to 72 h after challenge inoculation and the upregulation of *COI1* at 24 h after inoculation underscore the primacy of JA signaling in GL-induced resistance. Additionally, Arabidopsis mutants impaired in *JAR1* and *COI1* exhibited significantly higher susceptibility under GL compared to Col-0, reinforcing the critical role of the JA-mediated pathway in GL-induced resistance. Contrastingly, the SA-mediated defense pathway showed a delayed and less pronounced response to GL. Although *PAD4* expression increased at 0 and 24 h after inoculation in GL-treated plants, the upregulation of *SID2* and *PR1* occurred only 72 h after inoculation. This temporal distinction suggests that GL predominantly activates JA-dependent pathways during the early stages of infection, with SA-mediated defenses playing secondary and supportive roles. These findings highlight the complexity and specificity of GL-mediated signaling networks, suggesting that GL acts as a targeted modulator of plant immunity by differentially influencing JA- and SA-dependent pathways. GL illumination offers a unique advantage for fine-tuning plant defense. This study not only expands our understanding of light-regulated immunity but also opens new avenues for leveraging light spectra in precision agriculture.

## Discussion

Light is a fundamental environmental signal that not only drives plant growth and development but also shapes defense responses against pathogens. Although the effects of light wavelengths on plant photosynthesis and physiology are well documented, their role in modulating plant immunity at specific wavelengths remains a growing area of interest. Among the various light wavelengths, GL stands out as an intriguing yet underexplored player in plant‒pathogen interactions. Compared with RL and BL, GL is traditionally considered less efficient for photosynthesis because of its lower absorption by chlorophyll. However, GL increased leaf photosynthesis more efficiently than that by RL or BL in the lower chloroplasts under strong WL.^28^ Beyond its contribution to photosynthesis, GL contributes to plant defense by enhancing antioxidative enzyme activity and upregulating key photosynthetic genes, such as *LHCb* and *Psb*A.^29^ Furthermore, GL was reported to be effective in the control of various diseases, including leaf spot disease (caused by *Corynespora cassiicola*) in perilla, anthracnose in strawberry (*Glomerella cinglata*), anthracnose (*Colletotrichum orbiculare*), and gray mold (*Botrytis cinerea*) in cucumbers.^30^ Despite this potential, the molecular mechanisms and pathways underlying GL-mediated immunity remain largely unknown.

Light intensity and wavelength play crucial roles in determining plant defenses against pathogenic infections.^31^ Insufficient light can weaken plants, making them more susceptible to diseases, whereas excessive light intensity may cause photoinhibition, damaging the photosynthetic apparatus due to an imbalance between light absorption and utilization.^32^^,^^33^ High light treatment (100 µmol m^–2^ s^–1^) to Arabidopsis prior to PstDC3000 infection enhanced resistance, promoting local and systemic acquired resistance.^34^ In our earlier studies, GL illumination at an intensity of 100 µmol m^–2^ s^–1^ significantly reduced disease severity in tomato plants infected with *P. cichorii* JBC1.^18^ This study extends the understanding of the effects of monochromatic RL, GL, and BL on disease incidence in Arabidopsis Col-0. Our results revealed that Arabidopsis plants illuminated with monochromatic light (RL, GL, and BL) exhibited reduced disease severity compared with that by WL or darkness. GL at 100 µmol m^–2^ s^–1^ was effective in suppressing disease incidence. However, increasing the GL intensity to 125 µmol m^–2^ s^–1^ provided no additional benefits, suggesting a finely tuned optimal threshold for GL-mediated resistance. Arabidopsis Col-0 plants grown in complete darkness exhibited smaller leaves and early onset of disease symptoms, emphasizing the crucial role of light in plant immunity. Contrastingly, plants illuminated with BL and RL showed signs of stress, such as rough leaf textures and increased water absorption. These results indicate the wavelength-specific effects of light on plant physiology, revealing that different spectra can elicit distinct stress and defense responses.

The JA-mediated pathway is a cornerstone of plant resistance against necrotrophic pathogens, whereas the SA-mediated pathway is responsible for resistance against biotrophic pathogens such as PstDC3000. Here, GL illumination significantly upregulated key JA-dependent defense genes, including *JAR1, PDF1.2,* and *COI1*, immediately after challenge with PstDC3000. This is followed by the activation of SA-associated defense genes, such as *PR-1a,* at a later stage. These results suggest a phased response under GL, where JA signaling plays a dominant early role, complemented by subsequent SA activity. Contrastingly, under normal light conditions, van den Berg et al.^35^ reported the early expression of SA-mediated genes in an incompatible *Persea americana-Phytophthora cinnamomi* interaction, followed by JA-mediated gene expression. In our earlier studies, the expression of SA-dependent genes such as *PAL* and *PR-1a* was significantly upregulated in tomato plants challenged with *P. cichorii* JBC1 under GL illumination.^18^ Similarly, Zheng et al.^36^ reported the involvement of GL in the upregulation of SA levels in tea plants. Sato et al.^37^ observed a significant increase in both SA and JA levels under intermittent exposure to GL during the dark period in *A. thaliana*. Overall, our results indicate that GL regulates the expression of JA- and SA-mediated defense genes, with the JA-mediated pathway playing a prominent role in the early and effective response to PstDC3000 in Arabidopsis. Defense signals can interact synergistically or antagonistically, and their relative roles in plant defense may vary depending on environmental factors and the specific type of stress encountered,^38^ illustrating the multifaceted nature of plant immunity regulation.

The primacy of JA signaling in GL-induced resistance was further supported by the results from mutant Arabidopsis lines deficient in the JA (Δ*jar1,* Δ*coi1*) and SA (Δ*sid2,* Δ*npr1*) pathways. JA signaling-deficient mutants exhibited increased susceptibility under GL treatment. These results highlight the clear preferential activation of JA-related pathways in the early stages of infection, followed by a less pronounced activation of SA pathways at later stages. Taken together, these results indicate that GL-driven immunity may be orchestrated through a fine-tuned balance between these two pathways, with JA taking precedence in the initial responses before transitioning to an SA-mediated defense response.

GL also influences critical physical and oxidative defense mechanisms, as well as hormonal regulation. Although GL reduced H_2_O_2_ accumulation at later infection stages, it significantly enhanced callose deposition at the infection sites. Li et al.^39^ reported a similar reduction in ROS levels with increasing GL levels in melon seedlings under drought-stress conditions. ROS, which are central to plant defense, act as signaling molecules and antimicrobial agents. Earlier studies showed that light-induced ROS accumulation mediates systemic acquired acclimation.^40^^,^^41^ Light suppressed the *Pseudomonas syringae* pv. *tabaci* populations in tobacco leaves via accumulation of H_2_O_2_ during infection.^42^ The ability of GL to modulate ROS levels through JA signaling likely helps maintain redox balance and prevent oxidative damage while enhancing pathogen resistance. These results suggest that GL-mediated defense is primarily driven by JA signaling, with modulated ROS dynamics playing a complementary role. GL significantly enhanced uniform and extensive callose deposition at the infection sites, indicating a more robust and proactive defense response by GL than that by WL. Collectively, the analysis of H_2_O_2_ accumulation and callose deposition further supports the idea that GL-mediated resistance involves a finely tuned interplay between oxidative stress and defense activation.

Our findings suggest that the effects of GL on plant immunity are closely linked to a broad range of light factors. The optimal intensity and duration of GL exposure are crucial because both insufficient and excessive light can compromise plant health and immune responses.^32^^,^^33^ For example, under excessively high light conditions, plants may suffer from photoinhibition, which impairs their ability to effectively defend against pathogens. This highlights the dual role of light as both a potential enhancer and a stressor, depending on its managing technique. Striking the correct balance in GL exposure could emerge as a transformative strategy for optimizing crop resilience, particularly in pathogen-prone environments. Thus, managing light exposure could be a promising strategy for enhancing crop resilience as long as the balance between light intensity and plant health is carefully considered. Although this study reveals the importance of GL in activating JA-mediated defense mechanisms, it also raises compelling questions regarding the molecular mechanisms underlying GL perception and signaling in plants. Unlike UV, BL, and RL, whose photoreceptors and signaling cascades are well characterized, GL-specific photoreceptors and their downstream pathways remain elusive. The absence of identified GL-specific photoreceptors presents a significant knowledge gap holding the key to unlocking a deeper understanding of light-regulated immunity. Identifying the receptor(s) and unraveling their signaling cascades will be pivotal in understanding the orchestration of plant defenses by GL.

In conclusion, this study demonstrates that GL plays a pivotal role in enhancing plant resistance to pathogen infection through JA-mediated pathways, callose deposition, and ROS modulation. Our findings underscore the need to further investigate GL-specific photoreceptors and their downstream signaling networks, which remain largely unexplored. Identifying these receptors could open new avenues for the targeted modulation of plant immunity. We will further attempt to identify GL photoreceptors and their molecular networks, paving the way for innovative agricultural strategies to bolster plant resilience to pathogens.

## Supplementary Material

Supplementary data **Figure S1.** Light treatment system used in this study. **Figure S2**. Tentative diagram of green light-responsive disease resistance signaling networks. **Table S1.** Primers used for this study.

## Data Availability

All data generated or analyzed during this study were included in this manuscript.
