# Supplementary material for: Green light modulates disease resistance in Arabidopsis thaliana against Pseudomonas syringae pv. tomato
Source: Plant Signal Behav. 2025 Aug 20;20(1):2546465. doi: 10.1080/15592324.2025.2546465 (PMC12377085; doi:10.1080/15592324.2025.2546465)
Supplement: Supplementary data — Figure S1. Light treatment system used in this study. Figure S2. Tentative diagram of green light-responsive disease resistance signaling networks. Table S1. Primers used for this study. [file KPSB_A_2546465_SM9201.docx]

**Supplementary information**


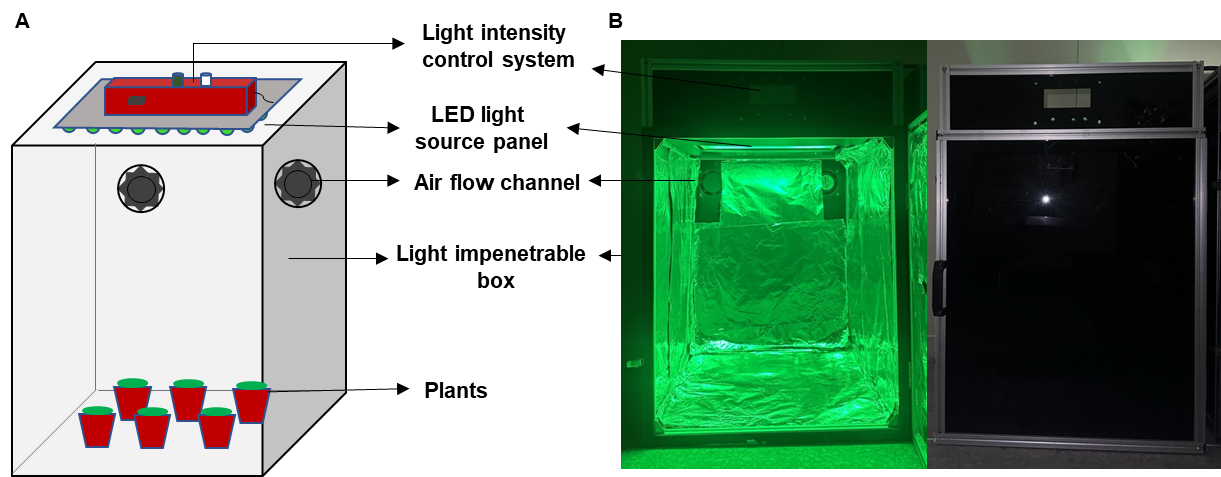


Fig. S1. Light treatment system used in this study. The LED sources emitting specific light wavelength, red (645 nm), green (524 nm), blue (458 nm), and white (broad spectrum) light, were installed in upper sealings of chambers. The LEDs were connected to a circuit box that allows the control of intensity for each wavelength. The chambers were equipped with air channels and enclosed with light impenetrable black walls to prevent external light interference. (A, B) Schematic diagram and actual images (opened and closed view) of the light treatment system used in the study, respectively.


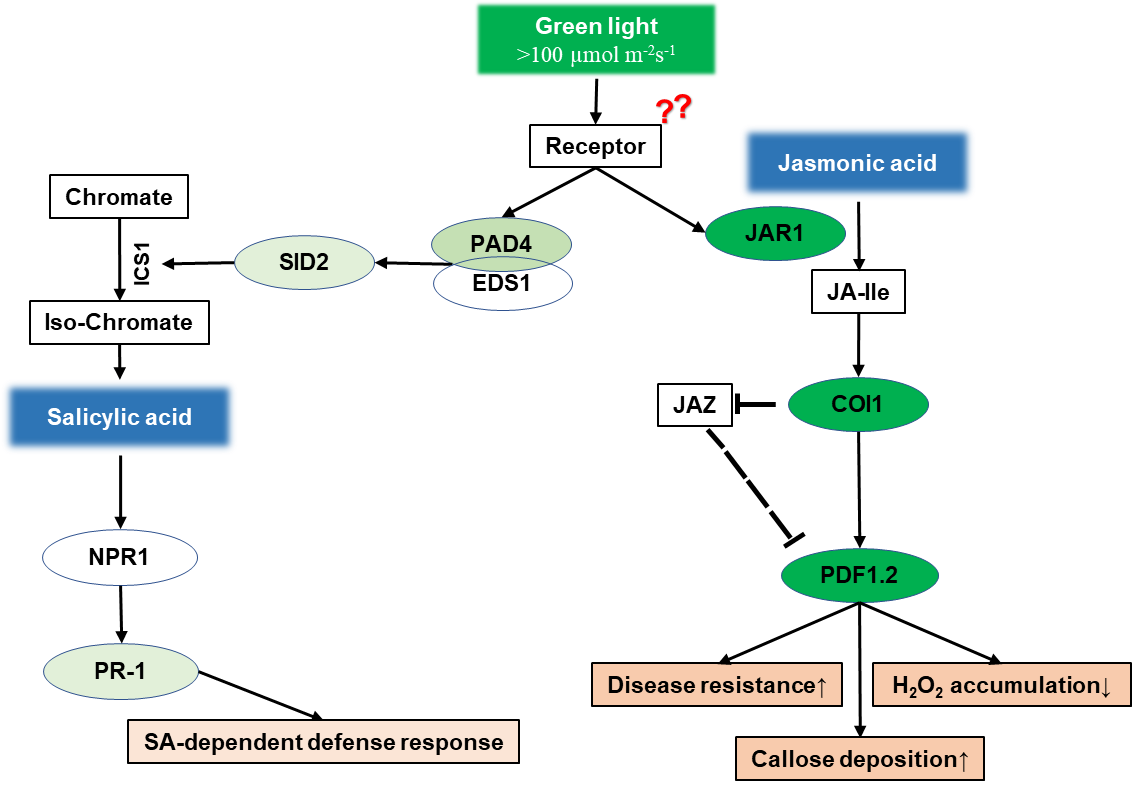


Fig. S2. Tentative diagram of green light-responsive disease resistance signaling networks. Green light (GL) mediates plant defense signaling mainly via JA-dependent pathway. It upregulates the expression of major JA-pathway genes including *JAR1, COI1,* and *PDF1.2*, while also inducing a minor increase in the expression of SA pathway genes such as *PAD4, SID2*, and *PR-1.* The upregulated genes are depicted in in green oval with darker green shades indicating higher expression levels. This highlights the critical role of GL in modulating plant defense signaling. Abbreviations: PAD4 (Phytoalexin Deficient 4), EDS1 (Enhanced Disease Susceptibility 1), SID2 (Salicylic Acid Induction Deficient 2), ICS1 (Isochorismate Synthase 1), NPR1 (Nonexpresser of Pathogenesis-Related Genes 1), PR1 (Pathogenesis-Related 1), JAR1 (Jasmonate-Resistant 1), COI1 (Coronatine-Insensitive 1), JAZ (Jasmonate ZIM-Domain Protein), PDF1.2 (Plant Defensin 1.2).

Table S1. Primers for used for this study

| Name | Primers (5’→3’) | Source |
| --- | --- | --- |
| *PR-1*-F  *PR-1*-R | 5’TCATACACTCTGGTGGGCCT 3’  5’ACCTCACTTTGGCACATCCG 3’ | This study |
| *JAR1-*F  *JAR1-*R | 5’ CGTTTCGTCTGATCGGGATG 3’  5’ CAGGGTCAGTAGCGTTTCCAT 3’ | This study |
| *PDF1.2-*F  *PDF1.2-*R | 5’ CCTTATCTTCGCTGCTCTTG 3’  5’ CACTTGTGTGCTGGGAAGAC 3’ | This study |
| *NPR1-*F  *NPR1-*R | 5’ GCCACTATGGCGGTTGAATG 3’  5’ CGTTGAGCAAGTGCAACTCT 3’ | This study |
| *SID2_F*  *SID2_R* | 5’ GAGACTTACGAAGGAAGATGATGAG 3’  5’ TGATCCCGACTGCAAATTCACTCTC 3’ | Chen et al. (2009)^1^ |
| *COI1_F*  *COI1_R* | 5’ GTGTCCTAATTTGGAAGTTCTCG 3’  5’ CTCCATTCCTTGTTCATCTGC 3’ | Huang et al. (2014)^2^ |
| *PAD4-F’*  *PAD4-R’* | 5’ GCCGGGATTACATACGTTGC 3’  5’ CCAATCCTTCCTTGATCTTTAACTG3’ | This study |
| *EDS1-F’*  *EDS1-R’* | 5’ CTCAAGCCAGCGATGAACAAG 3’  5’ TCTTGTGCTCACTCCAAGGTC 3’ | This study |
| *ACT2_cDNA_F*  *ACT2_cDNA_R* | 5’ AGTGGTCGTACAACCGGTATTGTG 3’  5’ CCGATCCAGACACTGTACTTCCTT 3’ | de Leone et al. (2020)^3^ |

**References**

1. Chen H, Xue L, Chintamanani S, Germain H, Lin H, Cui H, Cai R, Zuo J, Tang X, Li X, Guo H, Zhou Jm. Ethylene insensitive3 and ethylene insensitive3-like1 repress salicylic acid induction deficient2 expression to negatively regulate plant innate immunity in Arabidopsis. *Plant Cell*. 2009; 21:2527-40.
2. Huang H, Wang CL, Tian HX, Sun Y, Xie DX, Song SS. Amino acid substitutions of GLY98, LEU245 and GLU543 in COI1 distinctively affect jasmonate-regulated male fertility in Arabidopsis. *Sci China Life Sci*. 2014; 57: 145–154
3. de Leone MJ, Hernando CE, Romanowski A, Careno DA, Soverna AF, Sun H, Bologna NG, Vázquez M, Schneeberger K, Yanovsky MJ. Bacterial infection disrupts clock gene expression to attenuate immune responses. *Curr Biol*. 2020; 30:1740-1747.e6.
